# Use of Rapid Online Surveys to Assess People's Perceptions During Infectious Disease Outbreaks: A Cross-sectional Survey on COVID-19

**DOI:** 10.2196/18790

**Published:** 2020-04-02

**Authors:** Pascal Geldsetzer

**Affiliations:** 1 Division of Primary Care and Population Health Department of Medicine Stanford University Stanford, CA United States

**Keywords:** rapid online surveys, perceptions, knowledge, coronavirus, SARS-CoV-2, pandemic, infectious disease, outbreak, survey, COVID-19, public health

## Abstract

**Background:**

Given the extensive time needed to conduct a nationally representative household survey and the commonly low response rate of phone surveys, rapid online surveys may be a promising method to assess and track knowledge and perceptions among the general public during fast-moving infectious disease outbreaks.

**Objective:**

This study aimed to apply rapid online surveying to determine knowledge and perceptions of coronavirus disease 2019 (COVID-19) among the general public in the United States and the United Kingdom.

**Methods:**

An online questionnaire was administered to 3000 adults residing in the United States and 3000 adults residing in the United Kingdom who had registered with Prolific Academic to participate in online research. Prolific Academic established strata by age (18-27, 28-37, 38-47, 48-57, or ≥58 years), sex (male or female), and ethnicity (white, black or African American, Asian or Asian Indian, mixed, or “other”), as well as all permutations of these strata. The number of participants who could enroll in each of these strata was calculated to reflect the distribution in the US and UK general population. Enrollment into the survey within each stratum was on a first-come, first-served basis. Participants completed the questionnaire between February 23 and March 2, 2020.

**Results:**

A total of 2986 and 2988 adults residing in the United States and the United Kingdom, respectively, completed the questionnaire. Of those, 64.4% (1924/2986) of US participants and 51.5% (1540/2988) of UK participants had a tertiary education degree, 67.5% (2015/2986) of US participants had a total household income between US $20,000 and US $99,999, and 74.4% (2223/2988) of UK participants had a total household income between £15,000 and £74,999. US and UK participants’ median estimate for the probability of a fatal disease course among those infected with severe acute respiratory syndrome coronavirus 2 (SARS-CoV-2) was 5.0% (IQR 2.0%-15.0%) and 3.0% (IQR 2.0%-10.0%), respectively. Participants generally had good knowledge of the main mode of disease transmission and common symptoms of COVID-19. However, a substantial proportion of participants had misconceptions about how to prevent an infection and the recommended care-seeking behavior. For instance, 37.8% (95% CI 36.1%-39.6%) of US participants and 29.7% (95% CI 28.1%-31.4%) of UK participants thought that wearing a common surgical mask was “highly effective” in protecting them from acquiring COVID-19, and 25.6% (95% CI 24.1%-27.2%) of US participants and 29.6% (95% CI 28.0%-31.3%) of UK participants thought it was prudent to refrain from eating at Chinese restaurants. Around half (53.8%, 95% CI 52.1%-55.6%) of US participants and 39.1% (95% CI 37.4%-40.9%) of UK participants thought that children were at an especially high risk of death when infected with SARS-CoV-2.

**Conclusions:**

The distribution of participants by total household income and education followed approximately that of the US and UK general population. The findings from this online survey could guide information campaigns by public health authorities, clinicians, and the media. More broadly, rapid online surveys could be an important tool in tracking the public’s knowledge and misperceptions during rapidly moving infectious disease outbreaks.

## Introduction

When faced with rapidly moving infectious disease outbreaks, such as the coronavirus disease 2019 (COVID-19), assessing knowledge and perceptions of relevant populations has to be accomplished in a short time frame if the findings are to be informative to the public health response. Population-representative household surveys generally take many months of preparation and data collection [1]. Phone surveys are faster to conduct but have increasingly suffered from low response rates (typically well below 10% [2]), which can be a major source of bias even when extensive weighting adjustments are made [3]. In addition, unless they use interactive voice response (which tends to further decrease the response rate [4]), phone surveys require substantial human resources to conduct the interviews. Given these limitations, rapid online surveys, which demand minimal human resources (beyond those needed to design the questionnaire) and could reach large numbers of respondents in a short time frame, may be a valuable tool to assess (and monitor over time) knowledge and perceptions of an infectious disease in the midst of an outbreak.

COVID-19 was first reported in Wuhan, China, in December 2019 [5]. On March 11, 2020, the World Health Organization (WHO) declared COVID-19 a pandemic [6], and by March 17, 2020, there were more than 200,000 confirmed cases and over 8000 reported deaths from COVID-19 worldwide [7]. The course of the COVID-19 epidemic in the United States and the United Kingdom will likely be strongly impacted by how the population behaves, which is in turn influenced by what people know and believe about this disease [8]. A particular concern in this regard is the spread of dis- and misinformation about COVID-19 on social media sites, which has led the WHO to host a page with “myth busters” on their website and engage in discussions with social media companies [9]. Understanding what the general public knows about COVID-19 and which misperceptions they hold about the condition is important for US and UK public health authorities as well as the media to design effective information campaigns.

The speed with which COVID-19 is spreading across the world calls for rapid assessments of the population’s knowledge and perceptions of this infection [7]. This study tests a rapid online survey methodology to determine knowledge and misperceptions of COVID-19 among the general adult population in both the United States and the United Kingdom.

## Methods

### Sampling Participants

This is a cross-sectional online survey conducted on the research platform created and managed by Prolific Academic Ltd. Prolific is an online platform that connects researchers with individuals around the world who are interested in participating in online research studies [10]. The platform’s pool of participants consists of approximately 80,000 individuals, of whom about 43% reside in the United Kingdom and 33% reside in the United States [11]. Researchers are required to pay participants a minimum of US $6.50 per hour.

For this study, Prolific established strata by age group (18-27, 28-37, 38-47, 48-57, or ≥58 years), sex (male or female) and ethnicity (white, black or African American, Asian or Asian Indian, mixed, or “other”) as well as all combinations of these strata. Using numbers from the latest census in the United States and the United Kingdom, a given number of places for taking the questionnaire were opened on the Prolific platform in each stratum to achieve the same distribution of participants by age, sex, and ethnicity as those in the general population. The targeted total sample size in each country was 1500 people. Participants’ eligibility for the open places in a particular stratum was determined based on the information they had entered in their profile when registering with Prolific. Eligible participants enrolled in the study on a first-come, first-served basis. The study was implemented in two rounds of 1500 participants each in the United States and the United Kingdom, such that the total target sample size in each country was 3000. Participants had to have indicated that they are fluent in English when registering with Prolific to be eligible for this study.

### Data Collection

Data were collected using the online questionnaire shown in Multimedia Appendix 1. Participants received US $1.50 (equivalent to £1.17 on March 1, 2020) for completing the questionnaire. Following an informed consent form, the questionnaire asked participants about the cause, current state, and future development of the COVID-19 epidemic; the risk of a fatal disease course; knowledge of symptoms and recommended health care–seeking behavior; measures to prevent an infection with SARS-CoV-2; and their perception of the risk posed by individuals of East-Asian ethnicity in their community. In order to investigate to what degree dis- and misinformation about COVID-19 has affected the general public’s beliefs about the condition, participants were directly asked whether they believed several falsehoods listed on the WHO’s “myth busters” website [12], which the WHO selected because these myths were circulating on social media [13]. Specifically, the questionnaire asked whether receiving a letter or package from China poses a risk of infection and whether using hand dryers, rinsing your nose with saline, eating garlic, applying sesame oil to the skin, taking antibiotics, and vaccinating against pneumonia are effective in preventing a SARS-CoV-2 infection. The questionnaire was built using Qualtrics software. Participants had to answer a question to reach the next question. Numerical entry questions did not allow for nonsensical inputs (eg, percentage questions were restricted to inputs between 0 and 100).

### Data Analysis

For binary and categorical response options, the percentage of participants who selected each response was computed. For binomial proportions, two-sided 95% confidence intervals using the Wilson score interval were calculated [14]. No sampling weights were used, given that this was not a probabilistic sample of adults and that the survey was already self-weighting by the age, sex, and ethnicity groups used to establish the strata for sampling.

Three types of data quality checks were performed. First, participants who took less than 2 minutes to complete the questionnaire were excluded from the analysis because this indicated random clicking. This resulted in the exclusion of 2 participants. Second, if some respondents used random clicking to obtain the US $1.50 reward as fast as possible, a bimodal distribution in the time taken to complete the survey might be expected (with one group clicking as fast as possible and one reading the questions). I, therefore, plotted a histogram of the time taken to complete the survey. Third, participants were asked, at the end of the questionnaire, whether they looked up any answers online (*“It is natural to be tempted to look up the answer to a question, especially when it’s only a click away. For approximately how many of the questions did you first look up the answer on Google or somewhere else before responding? The answer to this question will not affect your payment in any way.”*) and if so, for which question. Those who self-reported looking up an answer online for a question were excluded from the analysis for that particular question. This was the case for 81 US participants and 63 UK participants who reported looking up the answer online for a median of 1 (IQR 1-5) and 1 (IQR 1-2.5) questions, respectively.

## Results

### Data Collection Time

**Figure 1:**
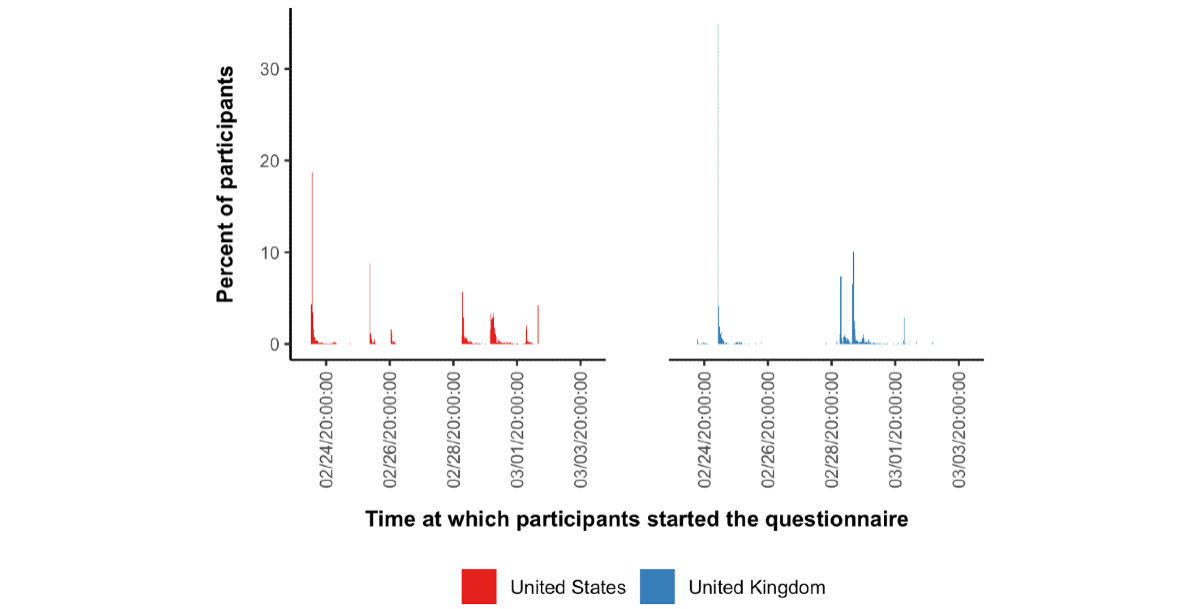
Time at which participants started the questionnaire. Dates and times are given in Pacific Standard Time. Bins have a width equal to 30 minutes.

Figure 1 shows that each of the two rounds of the survey took 2-3 days to conduct. There was no evidence of a bimodal distribution in the time taken to complete the survey (Multimedia Appendix 1).

### Participant Characteristics

Of 3000 adults residing in the United States and 3000 adults residing in the United Kingdom who could participate, 2986 and 2988, respectively, completed the questionnaire. Approximately two-thirds (1924/2986, 64.4%) of US participants and half (1540/2988, 51.5%) of UK participants had a tertiary education degree (Table 1). In addition, 67.5% (2015/2986) of US participants had a total household income between US $20,000 and US $99,999, and 74.4% (2223/2988) of UK participants had a total household income between £15,000 and £74,999. Furthermore, 17.3% (516/2986) of US participants and 13.7% (409/2988) of UK participants were currently students.

**Table 1:** Sample characteristics.

Characteristics		US	UK
Number of participants		2986	2988
Female, n (%)		1519 (50.9)	1531 (51.2)
**Age group (years), n (%)**			
	18-27	655 (21.9)	550 (18.4)
	28-37	687 (23.0)	557 (18.6)
	38-47	531 (17.8)	563 (18.8)
	48-57	493 (16.5)	480 (16.1)
	≥58	620 (20.8)	838 (28.0)
**Education, n (%)**			
	Less than a high school diploma/A-levels	24 (0.8)	396 (13.3)
	High school degree/Completed A-levels	334 (11.2)	682 (22.8)
	Some undergraduate education (no degree)	704 (23.6)	370 (12.4)
	Associate degree	322 (10.8)	N/A^a^
	Bachelor’s degree	1068 (35.8)	1030 (34.5)
	Master’s degree	405 (13.6)	330 (11.0)
	Professional degree	63 (2.1)	100 (3.3)
	Doctorate	66 (2.2)	80 (2.7)
**Total household income, n (%)**			
	<US $10,000/<£7500	165 (5.5)	172 (5.8)
	US $10,000-US $19,000/£7500-£14,999	222 (7.4)	333 (11.1)
	US $20,000-US $29,000/£15,000-£22,499	342 (11.5)	463 (15.5)
	US $30,000-US $39,000/£22,500 - £29,999	325 (10.9)	473 (15.8)
	US $40,000-US $49,000/£30,000-£37,499	280 (9.4)	358 (12.0)
	US $50,000-US $59,000/£37,500-£44,999	304 (10.2)	312 (10.4)
	US $60,000-US $69,000/£45,000-£52,499	230 (7.7)	242 (8.1)
	US $70,000-US $79,000/£52,500-£59,999	242 (8.1)	156 (5.2)
	US $80,000-US $89,000/£60,000-£67,499	138 (4.6)	121 (4.0)
	US $90,000-US $99,000/£67,500-£74,999	154 (5.2)	98 (3.3)
	US $100,000-US $149,000/£75,000-£99,999	401 (13.4)	168 (5.6)
	≥US $150,000/≥£100,000	183 (6.1)	92 (3.1)
**Race or ethnicity, n (%)**			
	White	2269 (76.0)	2540 (85.0)
	Black or African American	392 (13.1)	110 (3.7)
	Asian or Asian Indian	191 (6.4)	227 (7.6)
	Mixed	74 (2.5)	62 (2.1)
	Other	60 (2.0)	49 (1.6)
Current student, n (%)		516 (17.3)	409 (13.7)
**Chinese descent, n (%)**			
	Born in China	11 (0.4)	15 (0.5)
	Parents or grandparents born in China	57 (1.9)	27 (0.9)
**Works as a health care provider, n (%)**			
	Nurse	33 (1.1)	44 (1.5)
	Physician	5 (0.2)	15 (0.5)
	Pharmacist	10 (0.3)	6 (0.2)
	Other	102 (3.4)	118 (3.9)

^a^Associate degrees are not awarded in the UK.

### Cause, Current State, and Future Development of the COVID-19 Epidemic

**Figure 2:**
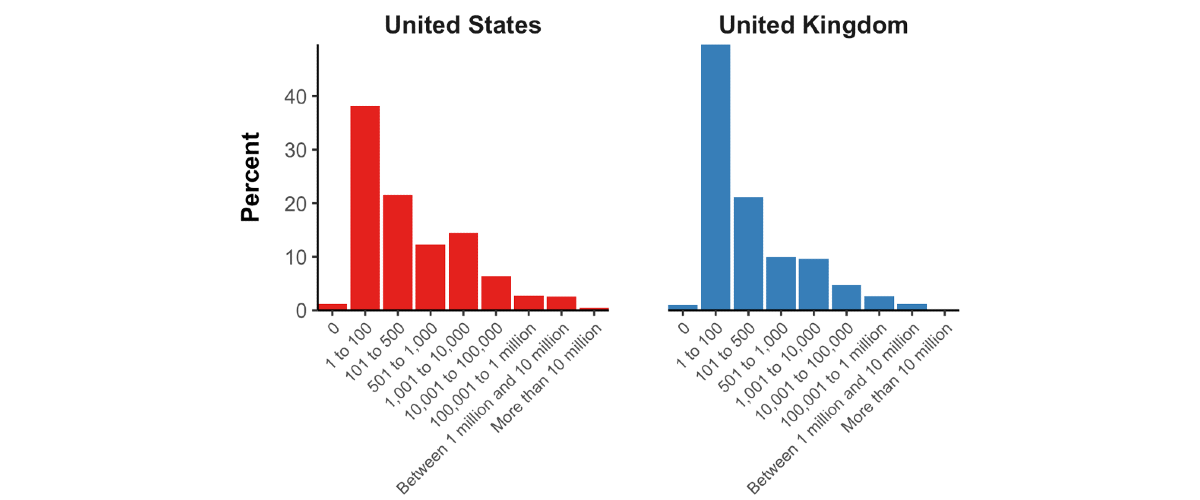
Proportion of participants who selected each category for their estimate of the number of COVID-19 deaths in their country by the end of 2020.

On a 7-point Likert scale ranging from “extremely unlikely” to “extremely likely,” 23.9% (95% CI 22.4%-25.5%) of US participants and 18.4% (95% CI 17.1%-19.9%) of UK participants selected “slightly likely,” “moderately likely,” or “extremely likely” when asked whether SARS-CoV-2 is a bioweapon developed by a government or terrorist organization (Multimedia Appendix 1). The US and UK participants estimated that a median of 100 (IQR 20-500) and 40 (IQR 13-200) individuals in their respective country were currently infected with COVID-19. In addition, a mean of 61.0% (95% CI 59.3%-62.8%) of US and 71.7% (95% CI 70.1%-73.3%) of UK participants thought that the number of fatalities from COVID-19 in their country will be ≤500 people by the end of 2020 (Figure 2).

### Case Fatality Rate

**Figure 3:**
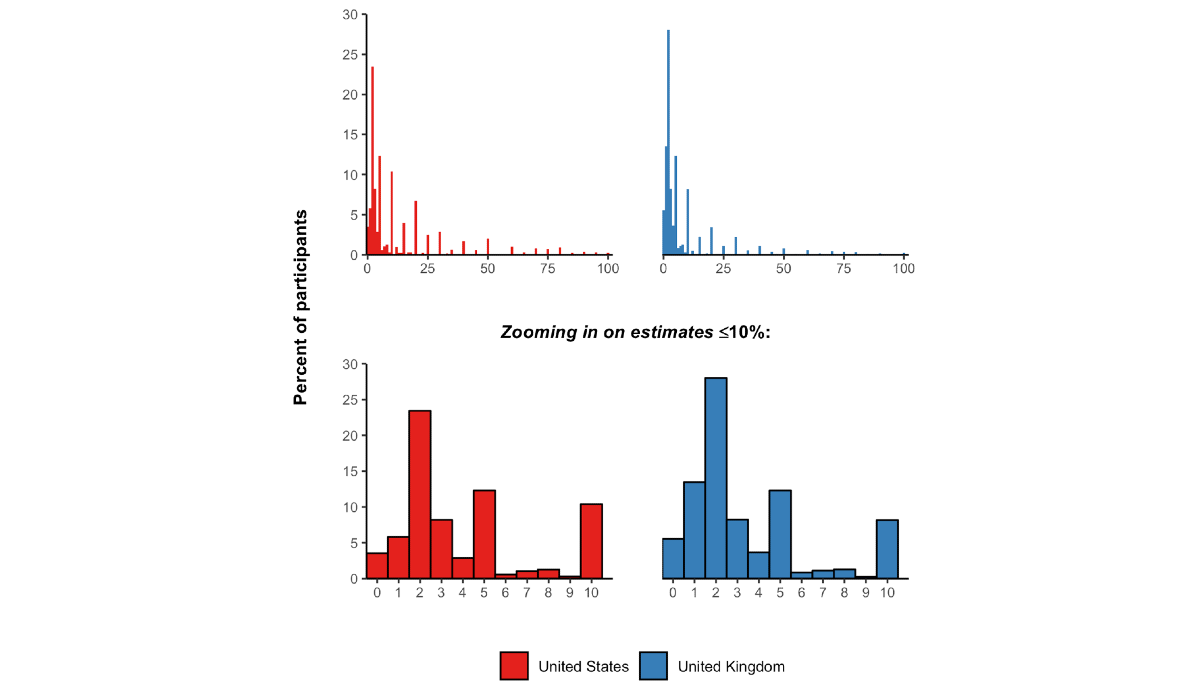
Distribution of responses to the question, “What percent of people who get infected with the new coronavirus die from this infection?”.

When asked what percent of individuals infected with COVID-19 experience a fatal disease course, the median estimate given by participants was 5.0% (IQR 2.0%-15.0%) among US participants and 3.0% (IQR 2.0%-10.0%) among UK participants. The full distribution of responses as well as a magnification of the distribution of responses among those who estimated a risk of death ≤10% is shown in Figure 3.

When asked “when they have been infected, what age groups are most likely to die from the illness caused by the new coronavirus” and presented with the option to select “children,” “young adults,” or “older adults” (selecting more than option was possible), 96.3% (95% CI 95.6%-96.9%) of participants in the United States and 98.3% (95% CI 97.7%-98.7%) of participants in the United Kingdom selected “older adults.” However, 53.8% (95% CI 52.1%-55.6%) in the United States and 39.1% (95% CI 37.4%-40.9%) in the United Kingdom also thought that children were at a high risk of death when infected. Almost all participants in both countries (96.3%, 95% CI 95.6%-97.0% in the United States and 97.5%, 95% CI 96.9%-98.0% in the United Kingdom) responded that adults with other health problems were more likely to experience a fatal disease course than those without any other health problems.

#### Symptoms of COVID-19 and Recommended Health Care–Seeking Behavior

**Figure 4:**
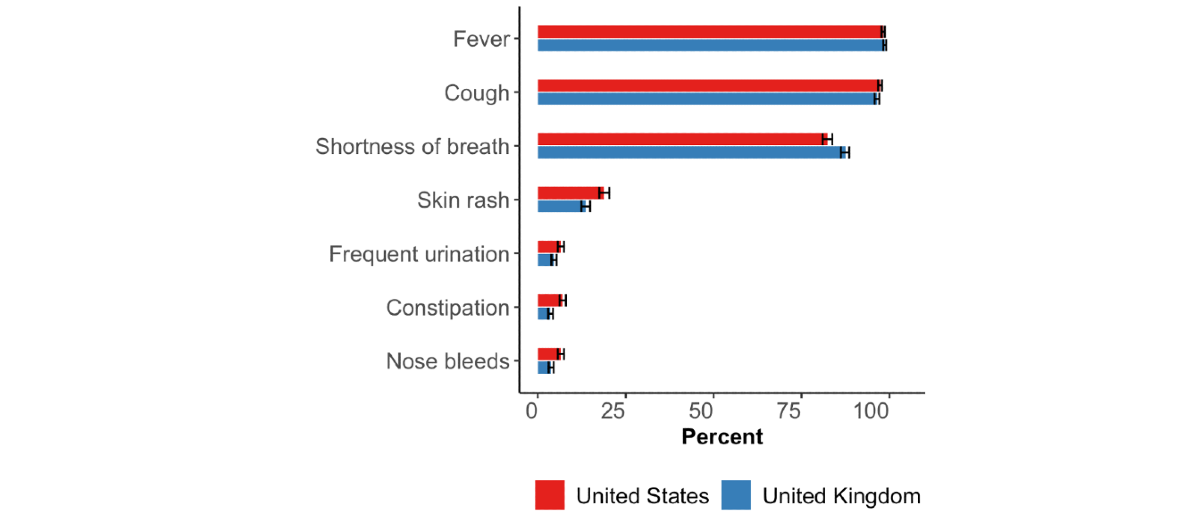
Proportion of participants who replied with “yes” to whether each of seven symptoms or signs were common for COVID-19. The horizontal black bars represent the 95% CIs calculated using the Wilson method [14].

Most participants in both the United States and the United Kingdom recognized fever, cough, and shortness of breath as three common symptoms and signs of COVID-19 (Figure 4).

**Figure 5:**
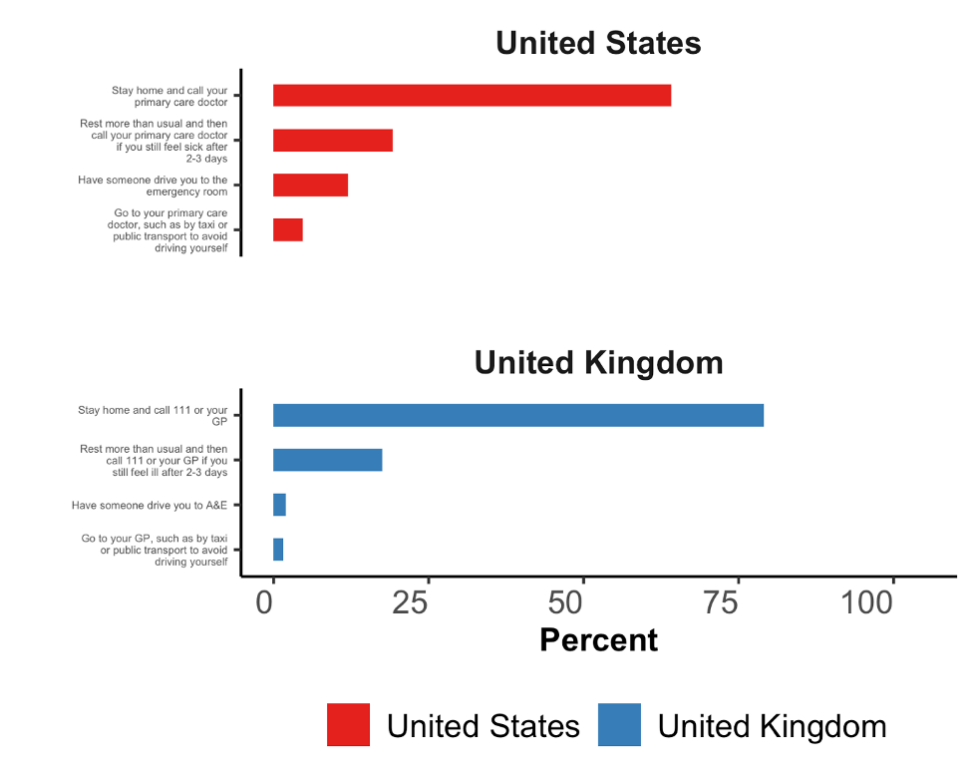
Responses to the question “If you have a fever or cough and recently visited China, or spent time with someone who did, what would be the best course of action?” GP: general practitioner; A&E: accident and emergency (department).

When asked *“if you have a fever or cough and recently visited China, or spent time with someone who did, what would be the best course of action?”* 64.2% (95% CI 62.4%-65.9%) of US participants and 79.0% (95% CI 77.5%-80.5%) of UK participants responded with the recommended care-seeking option of staying home and contacting their health system. About a third of respondents stated that they would either delay care-seeking, attend the hospital emergency department unannounced, or take a taxi or public transport to their primary care provider (Figure 5).

#### Preventing SARS-CoV-2 Infection

**Figure 6:**
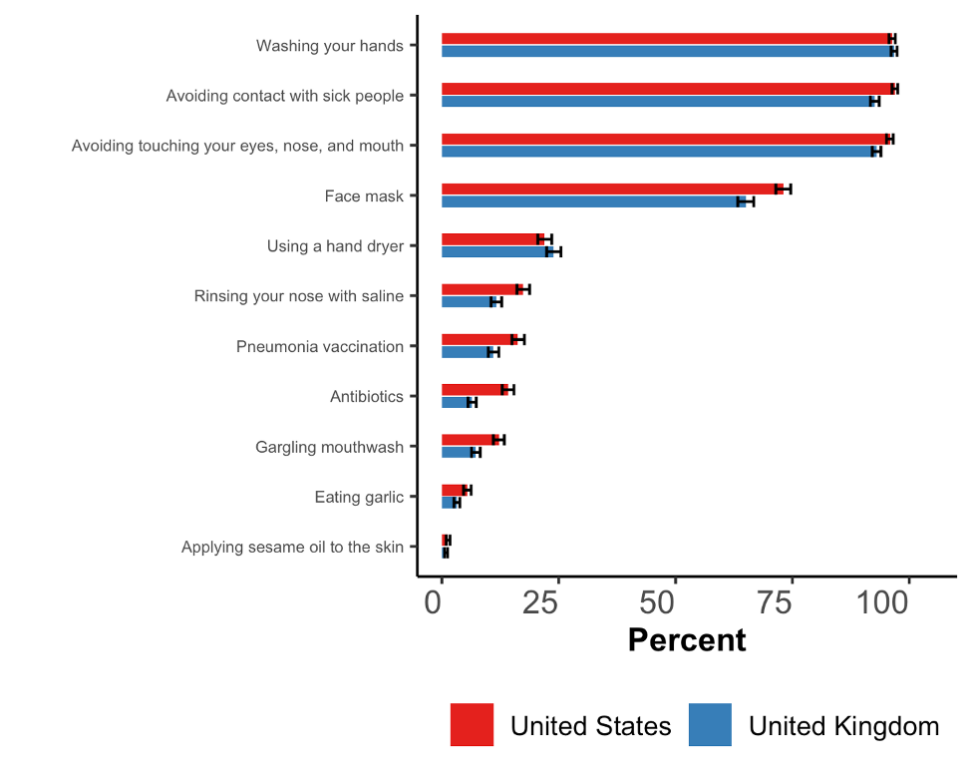
Proportion of participants who replied with “yes” to whether each of 11 actions help prevent an infection with SARS-CoV-2. The horizontal black bars represent the 95% CIs calculated using the Wilson method [14].

A total of 92.6% (95% CI 91.6%-93.4%) of US participants and 86.0% (95% CI 84.7%-87.2%) of UK participants selected each of the following three responses as effective measures for preventing infection with SARS-CoV-2: washing your hands; avoiding close contact with people who are sick; and avoiding touching your eyes, nose, and mouth with unwashed hands (Figure 6). However, a substantial proportion of participants also thought that using a hand dryer, rinsing your nose with saline, taking antibiotics, and gargling with mouthwash were effective prevention measures: 43.5% (95% CI 41.7%-45.2%) and 36.0% (95% CI 34.3%-37.8%) of US and UK participants, respectively, selected at least one of these options. Furthermore, 37.8% (95% CI 36.1%-39.6%) of US participants and 29.7% (95% CI 28.1%-31.4%) of UK participants agreed with the following statement: *“Consistently wearing a face mask is highly effective in protecting you from getting infected with the new coronavirus. For the purpose of this question, ‘highly effective’ is defined as reducing your risk of getting infected by >95% and a ‘face mask’ is a common medical mask.”*

**Figure 7:**
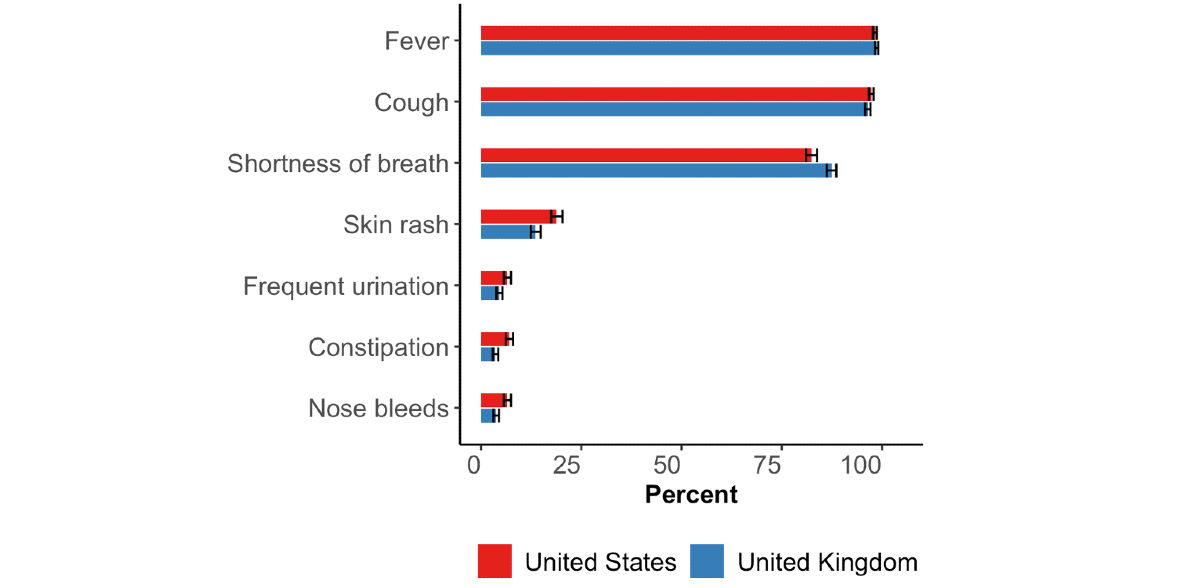
Proportion of participants who replied with “yes” to each government action in response to the question “At this point in the coronavirus epidemic, do you think your government should implement the following measures to prevent spreading of the virus?” The horizontal black bars represent the 95% CIs calculated using the Wilson method [14].

A total of 74.8% (95% CI 73.2%-76.4%) of US participants and 81.2% (95% CI 79.8%-82.6%) of UK participants correctly selected *“droplets of saliva that land in the mouths or noses of people who are nearby when an infected person sneezes or coughs”* as the main mode of SARS-CoV-2 transmission (Multimedia Appendix 1). Virtually all participants disagreed with the statement that *“only older adults can become infected with the new coronavirus”* (96.5%, 95% CI 95.8%-97.1% of US participants and 97.1%, 95% CI 96.5-97.7% of UK participants) and thought that there is currently no vaccine available that protects against COVID-19 (96.0%, 95% CI 95.3%-96.7% of US participants and 97.5%, 95% CI 96.9%-98.0% of UK participants). More than 20% of participants in both the United States and the United Kingdom thought that their government should quarantine everyone coming in from abroad for 14 days and suspend all air travel to their country (Figure 7).

#### Perceptions of the Risk Posed by Community Members of East-Asian Ethnicity

**Figure 8:**
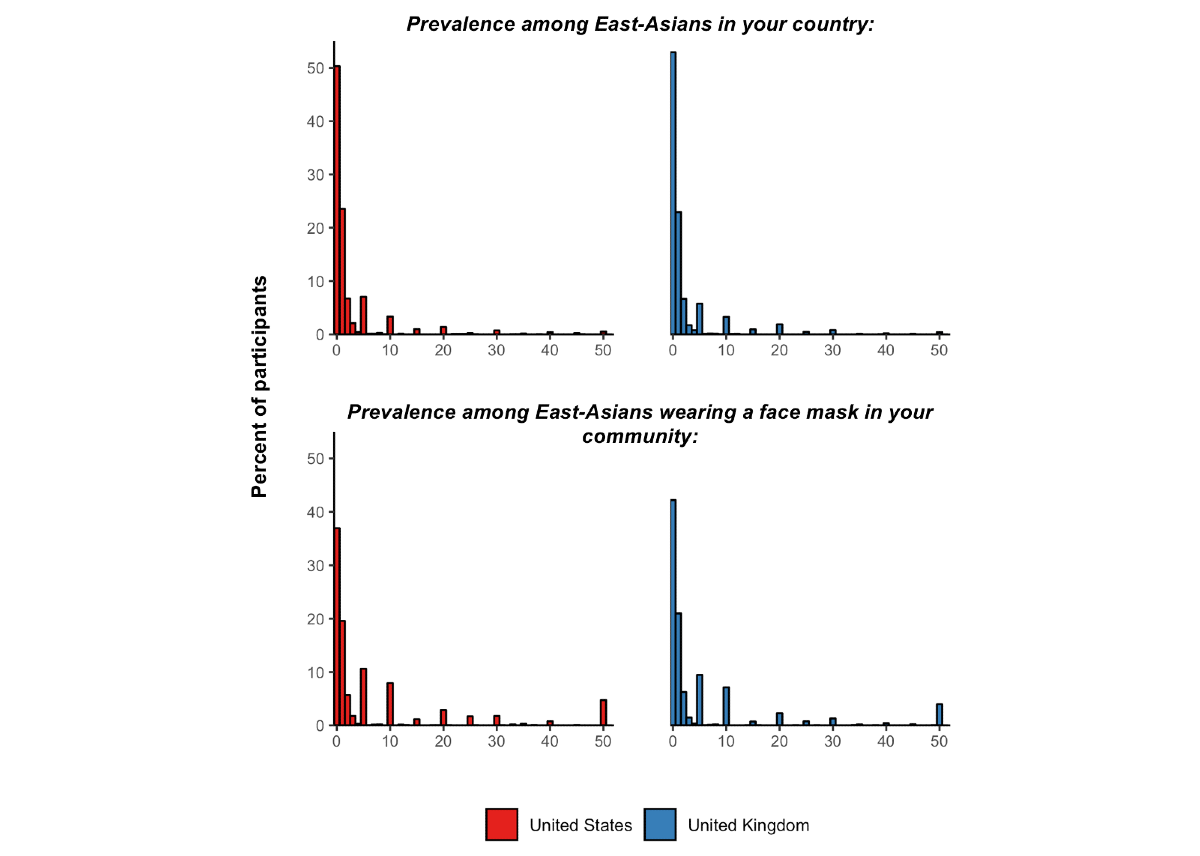
Distribution of the responses to questions on COVID-19 prevalence among individuals of East-Asian ethnicity. Of the total, 32 and 129 participants estimated a prevalence greater than 50% for the prevalence among East-Asian individuals in their country and East-Asian individuals wearing a face mask in their community, respectively. The responses from these individuals are not shown in the histogram below.

When asked about the prevalence of an infection with SARS-CoV-2 among East-Asian individuals in their country, the median estimate among US and UK participants was 0.5% (IQR 0.0%-2.0%) and 0.5% (IQR 0.0%-1.0%), respectively (Figure 8). The median increased to 1.0% (IQR 0.0%-5.0%) among both US and UK participants when asking about the prevalence of COVID-19 among *“adults of East-Asian ethnicity in your neighborhood who wear a face mask.”*

**Figure 9:**
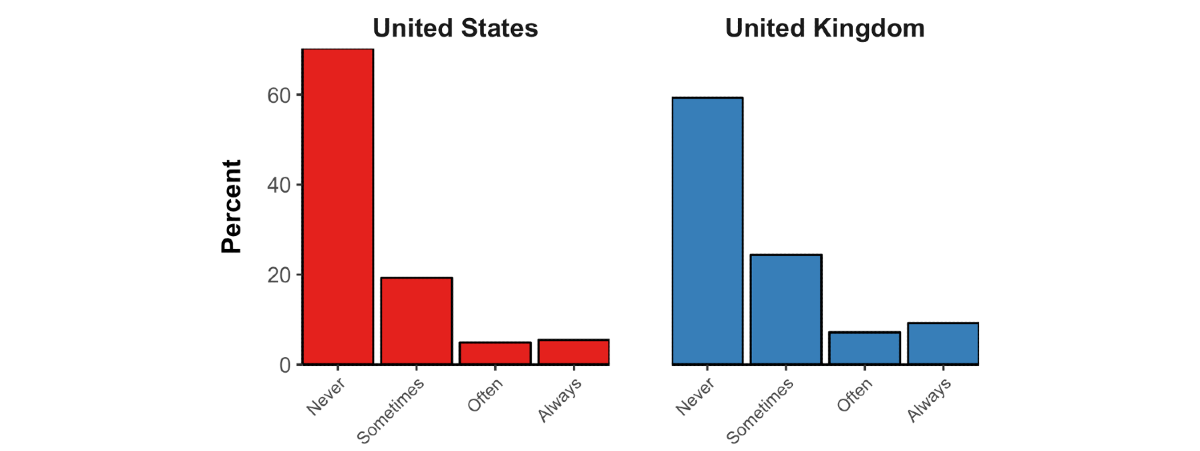
Distribution of responses to the question “If you were an Uber driver today, would you try to reject ride requests from people with East Asian-sounding names (or a profile photo of East-Asian ethnicity) to reduce your risk of getting infected with the new coronavirus?”.

A total of 25.6% (95% CI 24.1%-27.2%) of US participants and 29.6% (95% CI 28.0%-31.3%) of UK participants responded with “yes” to the question, *“Do you think it would be prudent for you to not eat at Chinese restaurants for the next few weeks to reduce the risk of getting infected with the new coronavirus?”* Approximately a quarter of participants (29.0%, 95% CI 27.4%- 30.7% of US participants and 24.4%, 95% CI 22.9%-26.0% of UK participants) thought one may become infected with SARS-CoV-2 by receiving a package from China. When asked *“If you were an Uber driver today, would you try to reject ride requests from people with East Asian-sounding names (or a profile photo of East-Asian ethnicity) to reduce your risk of getting infected with the new coronavirus?”* 29.7% (95% CI 28.1%-31.3%) of US participants and 40.8% (95% CI 39.0%-42.5%) of UK participants responded with “sometimes,” “often,” or “always” (Figure 9).

## Discussion

It was possible to conduct an in-depth online assessment of knowledge and perceptions of COVID-19 among the general public in the United States and the United Kingdom in a short time frame. It took 2-3 days to obtain a completed questionnaire of 22 knowledge and perception questions from 1500 adults in each the United States and the United Kingdom, when allowing enrollment only in relatively granular strata by age, sex, and ethnicity (and each of these variables’ combinations). Importantly, the distribution of participants by education and household income in this sample, although not part of the enrollment criteria, was similar to that of the general population in the United States and the United Kingdom [15,16]. In terms of data quality, there was no indication that participants randomly clicked on responses to earn the monetary reward as quickly as possible; only 2 participants took less than 2 minutes to complete the questionnaire and there was no evidence of a bimodal distribution in the time taken to complete the questionnaire. The direct cost to Prolific of administering the questionnaire was merely US $8961 (US $1.50 per completed questionnaire for a total of 5974 participants).

Regarding the survey findings, the general public in both the United States and the United Kingdom held several important misconceptions about COVID-19. Participants in both countries likely overestimated the probability of a fatal disease course among those infected with SARS-CoV-2 (while plagued by uncertainty, the case fatality rate is currently believed to lie below 1% [17]), thought that children were at an especially high risk of death from COVID-19 (which is currently not believed to be the case [5,17-19]), and believed that common surgical masks are highly effective in protecting them from SARS-CoV-2 infection. Participants also likely overestimated the prevalence of COVID-19 among East-Asian individuals in their communities. Likely as a result of this perception, a substantial proportion thought that they should refrain from frequenting Chinese restaurants, stated that they would refuse Uber rides to individuals of East-Asian ethnicity, and thought that receiving a package from China poses a risk of a SARS-CoV-2 infection. In general, differences in knowledge and misperceptions between US and UK participants were small.

This study’s findings on the levels of knowledge and prevalence of misconceptions regarding COVID-19 could inform relevant information campaigns by public health authorities and the media as well as communication of health care workers with patients. For instance, such information provision may highlight the comparatively low case fatality rate, the low risk posed by individuals of East-Asian ethnicity living in the United States and the United Kingdom, and that children do not appear to be at a heightened risk of dying from COVID-19. In addition, a substantial proportion of participants appeared to believe that common surgical masks are highly effective in protecting the wearer from infection with SARS-CoV-2. Information campaigns may, therefore, want to emphasize the comparative effectiveness of common surgical masks versus other methods of prevention, particularly frequent and thorough handwashing and avoiding close contact with people who are sick. Lastly, it is important to note that while the general public appeared to be well informed about the common symptoms of COVID-19, over one-fourth of the participants selected a health care–seeking option that could lead to further transmission of SARS-CoV-2. Thus, clear messaging on the recommended care-seeking action when experiencing some of the core symptoms of COVID-19 will be crucial.

Public health information campaigns may also want to directly target some of the mis- and disinformation that has circulated on social media [9,12,20]. Such measures could include information that rinsing your nose with saline, using a hand dryer, taking antibiotics, and gargling with mouthwash are not effective prevention measures and that receiving a letter or package from China does not pose a great risk of SARS-CoV-2 infection. These are all falsehoods listed on the WHO’s “myth busters” website, which a substantial proportion of participants in this study believed [12]. More broadly, this study underscores the need for the WHO and other public health bodies to continue working with social media campaigns to minimize the circulation of inaccurate information about COVID-19. In line with recent media reporting that this conspiracy theory has been actively spread on Twitter [20], about 1 in 5 participants believed it to be “slightly likely,” “moderately likely,” or “extremely likely” that SARS-CoV-2 is a bioweapon developed by a government or terrorist organization.

Participants did not expect a large number of individuals to die from COVID-19 in their country by the end of 2020. This finding may be surprising considering that fear-inducing headlines in the media may (at least up to a certain extent [21]) result in more attention by readers than more emotionally neutral ones, which could result in a catastrophizing of the epidemic. Moving forward, information campaigns on COVID-19 may need to balance the messaging of two important facts about the COVID-19 epidemic that could be interpreted by the general public to stand in direct conflict with each other: (1) The case fatality rate of COVID-19 appears to be lower than that of other recent infectious disease outbreaks such as Ebola infection [22], SARS [23], and the Middle East respiratory syndrome (MERS) [23,24]. On the other hand, however, the epidemic could cause a large number of fatalities, which implies that actions by governments and the general public to reduce transmission of SARS-CoV-2 could save many lives.

This study has several limitations. First, although the sample of participants is representative of the US and UK general population by age, sex, and ethnicity, and the distribution of participants by household income and education was similar to that in the US and UK general population, participants may still differ from the general population on a variety of other characteristics. These characteristics may be both correlated with their knowledge and perceptions of COVID-19 as well as with their decision to participate in the study or to create a profile with Prolific. Second, the estimates of discrimination against individuals of East-Asian ethnicity may be an underestimation because some participants may not have wanted to volunteer their discriminating tendencies to themselves or the researcher. However, I, as the researcher, had no access to any identifying information about the research participants, and participants were reminded of this fact prior to answering the question. In addition, such social desirability bias has been found to be lower in online surveys than in telephone or in-person surveys [25,26]. Third, it was possible for participants to randomly click responses in order to devote the least amount of time to earn the US $1.50 reward. In my view, this issue is unlikely to have caused major bias in this study because (1) there was no evidence of a bimodal distribution in the time taken to complete the survey (Multimedia Appendix 1); (2) while it was physically possible to complete the survey in under 90 seconds when randomly clicking on responses, only 2 participants completed the survey in under 2 minutes; and (3) the monetary reward (US $1.50) was relatively small, and thus, for most participants, unlikely to be the main motivation for participating in the study. Lastly, it is possible that individuals looked up the answers to some of the questions online prior to answering, which may have biased the results. Participants were reminded of the importance not to look up answers online prior to taking the survey and were asked at the end of the survey (while being reassured that their payment is not influenced by whether they volunteer information on having looked up an answer online) which, if any, questions they searched for an answer online prior to responding.

Rapid online surveys are a promising method to assess and track knowledge and perceptions in the midst of rapidly evolving infectious disease outbreaks. Such assessments are crucial because ensuring that the general public is well informed about a condition like COVID-19 could reduce unnecessary anxiety as well as reduce disease transmission and thus ultimately save lives.

## Appendix

Multimedia Appendix 1 Supplementary appendix.

## Abbreviations

COVID-19: coronavirus disease 2019
MERS: Middle East respiratory syndrome
SARS-CoV-2: severe acute respiratory syndrome coronavirus 2
WHO: World Health Organization
