# Supplementary material for: Use of Rapid Online Surveys to Assess People's Perceptions During Infectious Disease Outbreaks: A Cross-sectional Survey on COVID-19
Source: J Med Internet Res. 2020 Apr 2;22(4):e18790. doi: 10.2196/18790 (PMC7124956; doi:10.2196/18790)
Supplement: Multimedia Appendix 1 [file jmir_v22i4e18790_app1.docx]

**Supplementary appendix**

Text S1. Questionnaire used for participants residing in the United States 2

Text S2. Questionnaire used for participants residing in the United Kingdom 19

Figure S1. Distribution of the time taken (in seconds) to complete the survey 36

Figure S2. Distribution of responses to the question “Do you think it is likely that the new coronavirus is a bioweapon developed by a government or terrorist organization?” 37

Figure S3. Distribution of responses to the question “What is the main way in which people are currently getting infected with the new coronavirus? “ 38

# **Text S1. Questionnaire used for participants residing in the United States**

Start of Block: Informed Consent

Q1.1 **DESCRIPTION:** You are invited to participate in a research study on knowledge and perceptions that adults in the United States and the United Kingdom have of the coronavirus that first emerged in Wuhan, China, in 2019. You will be presented with questions on this coronavirus outbreak and the illness it causes. The objective of this research is to inform future public health efforts, particularly information campaigns for the general public. You will be asked to select options in multiple choice questions or type answers into text boxes.

**TIME INVOLVEMENT:** Your participation will take approximately 5 to 10 minutes.

**RISKS AND BENEFITS:** There are no foreseeable risks associated with this study. There are no direct benefits to you from participating in this study. However, the information you provide may help improve public health efforts with regards to the new coronavirus and, thus, reduce harm from this epidemic. We cannot and do not guarantee or promise that you will receive any benefits from this study.

**PAYMENTS:** You will receive $1.50 as payment for your participation.

**PARTICIPANT'S RIGHTS:**If you have read this form and have decided to participate in this project, please understand your participation is voluntary and you have the right to withdraw your consent or discontinue participation at any time without penalty or loss of benefits to which you are otherwise entitled. The alternative is not to participate. You have the right to refuse to answer particular questions. The results of this research study may be presented at scientific or professional meetings or published in scientific journals. Your individual privacy will be maintained in all published and written data resulting from the study.

**CONTACT INFORMATION:***Questions:* If you have any questions, concerns or complaints about this research, its procedures, risks and benefits, contact the Protocol Director, Dr. Pascal Geldsetzer at (+1) 415 694 8503. *Independent Contact:* If you are not satisfied with how this study is being conducted, or if you have any concerns, complaints, or general questions about the research or your rights as a participant, please contact the Stanford Institutional Review Board (IRB) to speak to someone independent of the research team at (+1) 650-723-2480 or email at IRB2-Manager@lists.stanford.edu, or toll free at 1-866-680-2906.  You can also write to the Stanford IRB, Stanford University, 1705 El Camino Real, Palo Alto, CA 94306. Please print a copy of this page for your records. If you agree to participate in this research, please select “I consent, begin the study” below.

- I consent, begin the study (1)
- I do not consent, I do not wish to participate (2)

End of Block: Informed Consent

Start of Block: Prolific ID

Q3.2 Before you start, please switch off your phone, e-mail, and music, so that you can focus on this study.


Thank you!


Please enter your Prolific ID here:

________________________________________________________________

End of Block: Prolific ID

Start of Block: Introduction

Q4.2 A new virus (of the coronavirus family) emerged in Wuhan, China, in 2019. We will refer to this virus from now on simply as “the new coronavirus”. We would love to find out what the general public in the United States knows about this new virus. 


For this research to be helpful to public health efforts, it is important that you refrain from looking up any answers online. A lot of aspects about this virus and the illness it causes are still unknown, so much of the time there is no correct answer. Please just give it your best guess. All you need to do to receive your payment is to fill out the answers to the best of your knowledge. 

End of Block: Introduction

Start of Block: Perceived risk of death from COVID-19

Q5.2 What percent of people who get infected with the new coronavirus die from this infection? No one knows the correct answer to this, so please just give it your best guess.
 
  *Please enter a number between 0 and 100. Decimals are allowed.*

________________________________________________________________

Q5.4 The current population of the United States is approximately 330 million people. How many people in the US do you think will die from the new coronavirus by the end of 2020?
 
*Please select only one option.*

- 0 (1)
- 1 – 100 (2)
- 101 – 500 (3)
- 501 – 1,000 (4)
- 1,001 – 10,000 (5)
- 10,001 – 100,000 (6)
- 100,001 – 1 million (7)
- Between 1 million and 10 million (8)
- Between 10 million and 100 million (9)
- More than 100 million (10)

Q5.6 When they have been infected, what age groups are most likely to die from the illness caused by the new coronavirus? 
  *Please select all options that you think are correct.*

- Children (1)
- Young adults (2)
- Older adults (3)

Q5.8 Are those with other health problems more likely to die from an infection with the new coronavirus disease than those without any other health problems?

- Yes (1)
- No (2)

Q77 What percent of people who get infected with the **common flu** end up dying from the common flu? 
  
 *Please enter a number between 0 and 100. Decimals are allowed.*

________________________________________________________________

End of Block: Perceived risk of death from COVID-19

Start of Block: Perception of risk posed by individuals of Asian ethnicity

Q6.2 When answering the next three questions, **please remember that your responses are completely anonymous**. We are not collecting nor asking for any identifying information, and Prolific never discloses to researchers any information associated with a given Prolific ID. 
 
Uber is a ride-sharing app that allows people to request and pay for a car ride via their phone. The Uber driver then either rejects or accepts the ride request. 


If you were an Uber driver today, would you try to reject ride requests from people with Asian-sounding names (or a profile photo of Asian ethnicity) to reduce your risk of getting infected with the new coronavirus?

- Always (1)
- Often (2)
- Sometimes (4)
- Never (5)

Q6.4 Again, please remember that your responses are completely anonymous. 
 
Do you think it would be prudent for you to not eat at Chinese restaurants for the next few weeks to reduce the risk of getting infected with the new coronavirus?

- Yes (1)
- No (2)

Q6.6 Suppose that you see an adult of Asian ethnicity in your neighborhood who wears a face mask. What do you think is the probability (in percent from 0% to 100%) that he or she is infected with the new coronavirus? 
 
*Please enter a number between 0 and 100. Decimals are allowed.*

________________________________________________________________

Q6.8 What percent of people of Asian ethnicity living in the United States do you think are currently infected with the new coronavirus? 
  *Please enter a number between 0 and 100. Decimals are allowed.*

________________________________________________________________

Q6.10 Does receiving a letter or package from China put you at risk of getting infected with the new coronavirus?

- Yes (1)
- No (2)

End of Block: Perception of risk posed by individuals of Asian ethnicity

Start of Block: Transmission of the new coronavirus

Q7.2 The population of the United States is approximately 330 million (330,000,000). What do you think is the number of people living in the United States who are infected with the new coronavirus? This number should include both those who have been diagnosed and those who have not been diagnosed.
 
*Please enter a number. Decimals are not allowed.*

________________________________________________________________

Q7.4 Only older adults can become infected with the new coronavirus.

- True (1)
- False (2)

Q7.6 Is there currently a vaccine available that protects against infection with the new coronavirus?

- Yes (1)
- No (2)

Q7.8 Which of the following actions help prevent catching an infection with the new coronavirus? 
 
*Please select 'True' or 'False' for each option.*

|  | True (1) | False (2) |
| --- | --- | --- |
| Wear a face mask (11) |  |  |
| Getting a vaccination against pneumonia (1) |  |  |
| Gargling mouthwash (2) |  |  |
| Washing your hands (3) |  |  |
| Eating garlic (4) |  |  |
| Avoid close contact with people who are sick (5) |  |  |
| Taking antibiotics (6) |  |  |
| Using a hand dryer (7) |  |  |
| Putting sesame oil on your skin (8) |  |  |
| Avoiding touching your eyes, nose, and mouth with unwashed hands (9) |  |  |
| Regularly rinsing your nose with saline (10) |  |  |

Q7.10 Is the following statement true or false? Consistently wearing a face mask is highly effective in protecting you from getting infected with the new coronavirus. 


For the purpose of this question, "highly effective" is defined as reducing your risk of getting infected by >95% and a "face mask" is a common medical mask.

- True (1)
- False (2)

Q7.12 What is the main way in which people are currently getting infected with the new coronavirus? 
*Please select one response option only.*

- Eating or touching bats (1)
- Fecal contaminants in drinking water (2)
- Unhygienic preparation of food (3)
- Sexual intercourse or sharing of needles for drug use (8)
- Mosquito bites (4)
- Droplets of saliva that land in the mouths or noses of people who are nearby when an infected person sneezes or coughs (5)
- Eating undercooked meat products (6)
- Directly coming into touch with someone's bodily fluids like blood, vomit, or sweat (7)
- Snake bites or touching snakes (10)

Q7.14 Approximately how far do you think the new coronavirus can travel through the air to transmit the infection from one person to another?
 
*Please give the number of feet. Decimals are allowed.*

________________________________________________________________

End of Block: Transmission of the new coronavirus

Start of Block: Recognizing and acting upon an infection

Q8.2 What are common signs or symptoms of an infection with the new coronavirus? 
  *Please select ‘True or ‘False’ for each option.*

|  | True (1) | False (2) |
| --- | --- | --- |
| Nose bleeds (1) |  |  |
| Cough (2) |  |  |
| Fever (3) |  |  |
| Skin rash (4) |  |  |
| Constipation (8) |  |  |
| Shortness of breath (5) |  |  |
| Frequent urination (7) |  |  |

Q8.4 If you have a fever or cough and recently visited China, or spent time with someone who did, what would be the best course of action? 
 
*Please select one response option only.*

- Go to your primary care doctor, such as by taxi or public transport to avoid driving yourself (3)
- Have someone drive you to the emergency room (1)
- Stay home and call your primary care doctor (2)
- Rest more than usual and then call your primary care doctor if you still feel sick after 2-3 days (4)

Q8.6 At this point in the coronavirus epidemic, do you think your government should implement the following measures to prevent spreading of the virus?

|  | Yes (1) | No (2) |
| --- | --- | --- |
| Quarantine everyone coming in from abroad for 14 days (3) |  |  |
| Suspend all air travel to your country (2) |  |  |
| Go door to door to measure everyone's temperature (4) |  |  |
| Close all schools (1) |  |  |
| Forbid any mass gatherings (e.g., sport events or concerts) (5) |  |  |
| Make it mandatory for adults to wear a face mask while outdoors (6) |  |  |
| Require everyone to remain in their home except to seek medical care and obtain food (7) |  |  |

Q8.8 What pieces of information about the new coronavirus do you feel the government should be providing you with, but has not yet done so? 
 
*Please type as many words as you would like into the text box below.*

________________________________________________________________

Q8.10 Do you think it is likely that the new coronavirus is a bioweapon developed by a government or terrorist organization?

- Extremely likely (1)
- Moderately likely (2)
- Slightly likely (3)
- Neither likely nor unlikely (4)
- Slightly unlikely (5)
- Moderately unlikely (6)
- Extremely unlikely (7)

Q8.12 It is natural to be tempted to look up the answer to a question, especially when it’s only a click away. For approximately how many of the questions above did you first look up the answer on Google or somewhere else before responding? The answer to this question will not affect your payment in any way.   
 
*Please enter a number below. Decimals are not allowed.*

________________________________________________________________

Display This Question:

If “If It is natural to be tempted to look up the answer to a question, especially when it’s only a click away. For approximately how many of the questions above did you first look up the answer on Google...” Text Response Is Greater Than 0

Q8.14 For which question(s) did you look up the answer on Google or somewhere else before responding? Again, the answer to this question will not affect your payment in any way.   

*Please select all questions that apply.*

- What percent of people who get infected with the new coronavirus die from this infection? (1)
- How many people in the US do you think will die from the new coronavirus by the end of 2020? (2)
- When they have been infected, what age groups are most likely to die from the illness caused by the new coronavirus? (3)
- Are those with other health problems more likely to die from an infection with the new coronavirus disease than those without any other health problems? (4)
- What percent of people who get infected with the common flu end up dying from the common flu? (20)
- If you were an Uber driver today, would you try to reject ride requests from people with Asian-sounding names to reduce your risk of getting infected with the new coronavirus? (15)
- Do you think it would be prudent for you to not eat at Chinese restaurants for the next few weeks to reduce the risk of getting infected with the new coronavirus? (16)
- Suppose that you see an adult of Asian ethnicity in your neighborhood who wears a face mask. What do you think is the probability (in percent from 0% to 100%) that he or she is infected with the new coronavirus? (17)
- What percent of people of Asian ethnicity living in the United States do you think are currently infected with the new coronavirus? (10)
- Does receiving a letter or package from China put you at risk of getting infected with the new coronavirus? (11)
- What do you think is the number of people living in the United States who are infected with the new coronavirus? (5)
- What is the main way in which people are currently getting infected with the new coronavirus? (21)
- Only older adults can become infected with the new coronavirus. True or false? (6)
- Is there currently a vaccine available that protects against infection with the new coronavirus? (7)
- Which of the following actions help prevent catching an infection with the new coronavirus? (8)
- What is the main way in which people are currently getting infected with the new coronavirus? (18)
- Approximately how far do you think the new coronavirus can travel through the air to transmit the infection from one person to another? (9)
- What are common signs or symptoms of an infection with the new coronavirus? (12)
- If you have a fever or cough and recently visited China, or spent time with someone who did, what would be the best course of action? (13)
- At this point in the coronavirus epidemic, do you think your government should implement the following measures to prevent spreading of the virus? (19)
- Do you think it is likely that the new coronavirus is a bioweapon developed by a government or terrorist organization? (14)

End of Block: Recognizing and acting upon an infection

Start of Block: Sociodemographics

Q9.2 Is your age 89 years or older?

- Yes (1)
- No (2)

Display This Question:

If Is your age 89 years or older? = No

Q9.4 What is your age?
 
*Please enter a number. Decimals are not allowed.*

________________________________________________________________

Q9.6 What is your gender?

- Male (1)
- Female (2)
- Other; please specify: (3) ________________________________________________

Q9.8 In which state do you currently reside?

State (1)

▼ Alabama (1) ... Wyoming ~ Weston County (3150)

Q9.10 What is the highest degree or level of school you have completed? (If you’re currently enrolled in school, please indicate the highest degree you have received.)

- Less than a high school diploma (1)
- High school degree or equivalent (e.g. GED) (2)
- Some college, no degree (3)
- Associate degree (e.g. AA, AS) (4)
- Bachelor’s degree (e.g. BA, BS) (5)
- Master’s degree (e.g. MA, MS, MEd) (6)
- Professional degree (e.g. MD, DDS, DVM) (7)
- Doctorate (e.g. PhD, EdD) (8)

Q9.12 Are you a healthcare provider, such as a nurse, physician, community health worker, or pharmacist?

- No, I'm not a healthcare provider (1)
- Nurse (2)
- Physician (3)
- Community health worker (4)
- Pharmacist (5)
- Other healthcare provider; please specify: (6) ________________________________________________

Q9.14 Were you, your parents, or your grandparents born in China? 
 
*Please select all options that apply.*

- No (1)
- I was born in China (2)
- At least one of my parents were born in China (3)
- At least one of my grandparents were born in China (4)

Q9.16 What is your race or ethnicity?   
 
*Please select all options that apply.*

- White (1)
- Hispanic, Latino, or Spanish (2)
- Black or African American (3)
- Asian or Asian Indian (4)
- American Indian or Alaska Native (5)
- Middle Eastern or North African (6)
- Native Hawaiian or Other Pacific Islander (7)
- Some other race or ethnicity; please specify: (8)

________________________________________________

Q9.18 What is your total household income?

- Less than $10,000 (1)
- $10,000 - $19,999 (2)
- $20,000 - $29,999 (3)
- $30,000 - $39,999 (4)
- $40,000 - $49,999 (5)
- $50,000 - $59,999 (6)
- $60,000 - $69,999 (7)
- $70,000 - $79,999 (8)
- $80,000 - $89,999 (9)
- $90,000 - $99,999 (10)
- $100,000 - $149,999 (11)
- $150,000 or more (12)

End of Block: Sociodemographics

# **Text S2. Questionnaire used for participants residing in the United Kingdom**

Start of Block: Informed Consent

Q1.1 **DESCRIPTION:** You are invited to participate in a research study on knowledge and perceptions that adults in the United States and the United Kingdom have of the coronavirus that first emerged in Wuhan, China, in 2019. You will be presented with questions on this coronavirus outbreak and the illness it causes. The objective of this research is to inform future public health efforts, particularly information campaigns for the general public. You will be asked to select options in multiple choice questions or type answers into text boxes.

**TIME INVOLVEMENT:** Your participation will take approximately 5 to 10 minutes.

**RISKS AND BENEFITS:** There are no foreseeable risks associated with this study. There are no direct benefits to you from participating in this study. However, the information you provide may help improve public health efforts with regards to the new coronavirus and, thus, reduce harm from this epidemic. We cannot and do not guarantee or promise that you will receive any benefits from this study.

**PAYMENTS:** You will receive $1.50 as payment for your participation.

**PARTICIPANT'S RIGHTS:**If you have read this form and have decided to participate in this project, please understand your participation is voluntary and you have the right to withdraw your consent or discontinue participation at any time without penalty or loss of benefits to which you are otherwise entitled. The alternative is not to participate. You have the right to refuse to answer particular questions. The results of this research study may be presented at scientific or professional meetings or published in scientific journals. Your individual privacy will be maintained in all published and written data resulting from the study.

**CONTACT INFORMATION:***Questions:* If you have any questions, concerns or complaints about this research, its procedures, risks and benefits, contact the Protocol Director, Dr. Pascal Geldsetzer at (+1) 415 694 8503. *Independent Contact:* If you are not satisfied with how this study is being conducted, or if you have any concerns, complaints, or general questions about the research or your rights as a participant, please contact the Stanford Institutional Review Board (IRB) to speak to someone independent of the research team at (+1) 650-723-2480 or email at IRB2-Manager@lists.stanford.edu, or toll free at 1-866-680-2906.  You can also write to the Stanford IRB, Stanford University, 1705 El Camino Real, Palo Alto, CA 94306. Please print a copy of this page for your records. If you agree to participate in this research, please select “I consent, begin the study” below.

- I consent, begin the study (1)
- I do not consent, I do not wish to participate (2)

End of Block: Informed Consent

Start of Block: Prolific ID

Q3.2 Before you start, please switch off your phone, e-mail, and music, so that you can focus on this study.


Thank you!


Please enter your Prolific ID here:

________________________________________________________________

End of Block: Prolific ID

Start of Block: Introduction

Q4.2 A new virus (of the coronavirus family) emerged in Wuhan, China, in 2019. We will refer to this virus from now on simply as “the new coronavirus”. We would love to find out what the general public in the United Kingdom knows about this new virus. 


For this research to be helpful to public health efforts, it is important that you refrain from looking up any answers online. A lot of aspects about this virus and the illness it causes are still unknown, so much of the time there is no correct answer. Please just give it your best guess. All you need to do to receive your payment is to fill out the answers to the best of your knowledge. 

End of Block: Introduction

Start of Block: Perceived risk of death from COVID-19

Q5.2 What percent of people who get infected with the new coronavirus die from this infection? No one knows the correct answer to this, so please just give it your best guess.
 
  *Please enter a number between 0 and 100. Decimals are allowed.*

________________________________________________________________

Q5.4 The current population of the UK is approximately 68 million people. How many people in the UK do you think will die from the new coronavirus by the end of 2020?
 
*Please select only one option.*

- 0 (1)
- 1 – 100 (2)
- 101 – 500 (3)
- 501 – 1,000 (4)
- 1,001 – 10,000 (5)
- 10,001 – 100,000 (6)
- 100,001 – 1 million (7)
- Between 1 million and 10 million (8)
- Between 10 million and 30 million (9)
- More than 30 million (10)

Q5.6 When they have been infected, what age groups are most likely to die from the illness caused by the new coronavirus? 

*Please select all options that you think are correct.*

- Children (1)
- Young adults (2)
- Older adults (3)

Q5.8 Are those with other health problems more likely to die from an infection with the new coronavirus disease than those without any other health problems?

- Yes (1)
- No (2)

Q77 What percent of people who get infected with the **common flu** end up dying from the common flu? 
  
 *Please enter a number between 0 and 100. Decimals are allowed.*

________________________________________________________________

End of Block: Perceived risk of death from COVID-19

Start of Block: Perception of risk posed by individuals of Asian ethnicity

Q6.2 When answering the next three questions, **please remember that your responses are completely anonymous**. We are not collecting nor asking for any identifying information, and Prolific never discloses to researchers any information associated with a given Prolific ID. 
 
Uber is a ride-sharing app that allows people to request and pay for a car ride via their phone. The Uber driver then either rejects or accepts the ride request. 


If you were an Uber driver today, would you try to reject ride requests from people with East Asian-sounding names (or a profile photo of East-Asian ethnicity) to reduce your risk of getting infected with the new coronavirus?

- Always (1)
- Often (2)
- Sometimes (4)
- Never (5)

Q6.4 Again, please remember that your responses are completely anonymous. 
 
Do you think it would be prudent for you to not eat at Chinese restaurants for the next few weeks to reduce the risk of getting infected with the new coronavirus?

- Yes (1)
- No (2)

Q6.6 Suppose that you see an adult of East-Asian ethnicity in your neighborhood who wears a face mask. What do you think is the probability (in percent from 0% to 100%) that he or she is infected with the new coronavirus? 
 
*Please enter a number between 0 and 100. Decimals are allowed.*

________________________________________________________________

Q6.8 What percent of people of East-Asian ethnicity living in the United Kingdom do you think are currently infected with the new coronavirus? 
  *Please enter a number between 0 and 100. Decimals are allowed.*

________________________________________________________________

Q6.10 Does receiving a letter or package from China put you at risk of getting infected with the new coronavirus?

- Yes (1)
- No (2)

End of Block: Perception of risk posed by individuals of Asian ethnicity

Start of Block: Transmission of the new coronavirus

Q7.2 The population of the United Kingdom is approximately 68 million (68,000,000). What do you think is the number of people living in the United Kingdom who are infected with the new coronavirus? This number should include both those who have been diagnosed and those who have not been diagnosed.
 
*Please enter a number. Decimals are not allowed.*

________________________________________________________________

Q7.4 Only older adults can become infected with the new coronavirus.

- True (1)
- False (2)

Q7.6 Is there currently a vaccine available that protects against infection with the new coronavirus?

- Yes (1)
- No (2)

Q7.8 Which of the following actions help prevent catching an infection with the new coronavirus? 
 
*Please select 'True' or 'False' for each option.*

|  | True (1) | False (2) |
| --- | --- | --- |
| Wear a face mask (11) |  |  |
| Getting a vaccination against pneumonia (1) |  |  |
| Gargling mouthwash (2) |  |  |
| Washing your hands (3) |  |  |
| Eating garlic (4) |  |  |
| Avoid close contact with people who are sick (5) |  |  |
| Taking antibiotics (6) |  |  |
| Using a hand dryer (7) |  |  |
| Putting sesame oil on your skin (8) |  |  |
| Avoiding touching your eyes, nose, and mouth with unwashed hands (9) |  |  |
| Regularly rinsing your nose with saline (10) |  |  |

Q7.10 Is the following statement true or false? Consistently wearing a face mask is highly effective in protecting you from getting infected with the new coronavirus. 


For the purpose of this question, "highly effective" is defined as reducing your risk of getting infected by >95% and a "face mask" is a common medical mask.

- True (1)
- False (2)

Q7.12 What is the main way in which people are currently getting infected with the new coronavirus? 
*Please select one response option only.*

- Eating or touching bats (1)
- Fecal contaminants in drinking water (2)
- Unhygienic preparation of food (3)
- Sexual intercourse or sharing of needles for drug use (8)
- Mosquito bites (4)
- Droplets of saliva that land in the mouths or noses of people who are nearby when an infected person sneezes or coughs (5)
- Eating undercooked meat products (6)
- Directly coming into touch with someone's bodily fluids like blood, vomit, or sweat (7)
- Snake bites or touching snakes (10)

Q7.14 Approximately how far do you think the new coronavirus can travel through the air to transmit the infection from one person to another?
 
*Please give the number of feet. Decimals are allowed.*

________________________________________________________________

End of Block: Transmission of the new coronavirus

Start of Block: Recognizing and acting upon an infection

Q8.2 What are common signs or symptoms of an infection with the new coronavirus? 
  *Please select ‘True or ‘False’ for each option.*

|  | True (1) | False (2) |
| --- | --- | --- |
| Nose bleeds (1) |  |  |
| Cough (2) |  |  |
| Fever (3) |  |  |
| Skin rash (4) |  |  |
| Constipation (8) |  |  |
| Shortness of breath (5) |  |  |
| Frequent urination (7) |  |  |

Q8.4 If you have a fever or cough and recently visited China, or spent time with someone who did, what would be the best course of action? 
 
*Please select one response option only.*

- Go to your General Practitioner (GP), such as by taxi or public transport to avoid driving yourself (3)
- Have someone drive you to the Accident & Emergency (A&E) department (1)
- Stay home and call 111 (NHS Direct/NHS 24/NHS Direct Wales) or your General Practitioner (GP) (2)
- Rest more than usual and then call 111 (NHS Direct/NHS 24/NHS Direct Wales) or your General Practitioner (GP) if you still feel ill after 2-3 days (4)

Q8.6 At this point in the coronavirus epidemic, do you think your government should implement the following measures to prevent spreading of the virus?

|  | Yes (1) | No (2) |
| --- | --- | --- |
| Quarantine everyone coming in from abroad for 14 days (3) |  |  |
| Suspend all air travel to your country (2) |  |  |
| Go door to door to measure everyone's temperature (4) |  |  |
| Close all schools (1) |  |  |
| Forbid any mass gatherings (e.g., sport events or concerts) (5) |  |  |
| Make it mandatory for adults to wear a face mask while outdoors (6) |  |  |
| Require everyone to remain in their home except to seek medical care and obtain food (7) |  |  |

Q8.8 What pieces of information about the new coronavirus do you feel the government should be providing you with, but has not yet done so? 
 
*Please type as many words as you would like into the text box below.*

________________________________________________________________

Q8.10 Do you think it is likely that the new coronavirus is a bioweapon developed by a government or terrorist organization?

- Extremely likely (1)
- Moderately likely (2)
- Slightly likely (3)
- Neither likely nor unlikely (4)
- Slightly unlikely (5)
- Moderately unlikely (6)
- Extremely unlikely (7)

Q8.12 It is natural to be tempted to look up the answer to a question, especially when it’s only a click away. For approximately how many of the questions above did you first look up the answer on Google or somewhere else before responding? The answer to this question will not affect your payment in any way.   
 
*Please enter a number below. Decimals are not allowed.*

________________________________________________________________

Display This Question:

If “If It is natural to be tempted to look up the answer to a question, especially when it’s only a click away. For approximately how many of the questions above did you first look up the answer on Google...” Text Response Is Greater Than 0

Q8.14 For which question(s) did you look up the answer on Google or somewhere else before responding? Again, the answer to this question will not affect your payment in any way.   

*Please select all questions that apply.*

- What percent of people who get infected with the new coronavirus die from this infection? (1)
- How many people in the UK do you think will die from the new coronavirus by the end of 2020? (2)
- When they have been infected, what age groups are most likely to die from the illness caused by the new coronavirus? (3)
- Are those with other health problems more likely to die from an infection with the new coronavirus disease than those without any other health problems? (4)
- What percent of people who get infected with the common flu end up dying from the common flu? (20)
- If you were an Uber driver today, would you try to reject ride requests from people with East Asian-sounding names to reduce your risk of getting infected with the new coronavirus? (15)
- Do you think it would be prudent for you to not eat at Chinese restaurants for the next few weeks to reduce the risk of getting infected with the new coronavirus? (16)
- Suppose that you see an adult of East-Asian ethnicity in your neighborhood who wears a face mask. What do you think is the probability (in percent from 0% to 100%) that he or she is infected with the new coronavirus? (17)
- What percent of people of East-Asian ethnicity living in the United States do you think are currently infected with the new coronavirus? (10)
- Does receiving a letter or package from China put you at risk of getting infected with the new coronavirus? (11)
- What do you think is the number of people living in the United Kingdom who are infected with the new coronavirus? (5)
- What is the main way in which people are currently getting infected with the new coronavirus? (21)
- Only older adults can become infected with the new coronavirus. True or false? (6)
- Is there currently a vaccine available that protects against infection with the new coronavirus? (7)
- Which of the following actions help prevent catching an infection with the new coronavirus? (8)
- What is the main way in which people are currently getting infected with the new coronavirus? (18)
- Approximately how far do you think the new coronavirus can travel through the air to transmit the infection from one person to another? (9)
- What are common signs or symptoms of an infection with the new coronavirus? (12)
- If you have a fever or cough and recently visited China, or spent time with someone who did, what would be the best course of action? (13)
- At this point in the coronavirus epidemic, do you think your government should implement the following measures to prevent spreading of the virus? (19)
- Do you think it is likely that the new coronavirus is a bioweapon developed by a government or terrorist organization? (14)

End of Block: Recognizing and acting upon an infection

Start of Block: Sociodemographics

Q9.2 Is your age 89 years or older?

- Yes (1)
- No (2)

Display This Question:

If Is your age 89 years or older? = No

Q9.4 What is your age?
 
*Please enter a number. Decimals are not allowed.*

________________________________________________________________

Q9.6 What is your gender?

- Male (1)
- Female (2)
- Other; please specify: (3) ________________________________________________

Q9.8 In which part of the UK do you currently reside?

- England (1)
- Scotland (2)
- Wales (3)
- Northern Ireland (4)

Q9.10 What is the highest degree or level of school you have completed? (If you’re currently enrolled in school, please indicate the highest degree you have received.)

- Less than A Levels (1)
- A Levels or equivalent (2)
- Some undergraduate university education, no degree (3)
- Bachelor’s degree (e.g. BA, BSc) (5)
- Master’s degree (e.g. MSc) (6)
- Professional degree (e.g. MBBS) (7)
- Doctorate (e.g. PhD) (8)

Q9.12 Are you a healthcare provider, such as a nurse, physician, community health worker, or pharmacist?

- No, I'm not a healthcare provider (1)
- Nurse (2)
- Physician (3)
- Community health worker (4)
- Pharmacist (5)
- Other healthcare provider; please specify: (6) ________________________________________________

Q9.14 Were you, your parents, or your grandparents born in China? 
 
*Please select all options that apply.*

- No (1)
- I was born in China (2)
- At least one of my parents were born in China (3)
- At least one of my grandparents were born in China (4)

Q9.16 What is your race or ethnicity?   
 
*Please select all options that apply.*

- White (1)
- Hispanic, Latino, or Spanish (2)
- Black or African American (3)
- Asian or Asian Indian (4)
- American Indian or Alaska Native (5)
- Middle Eastern or North African (6)
- Native Hawaiian or Other Pacific Islander (7)
- Some other race or ethnicity; please specify: (8)

________________________________________________

Q9.18 What is your total household income?

- Less than GBP 7,500 (1)
- GBP 7,500 - GBP 14,999 (2)
- GBP 15,000 - GBP 22,499 (3)
- GBP 22,500 - GBP 29,999 (4)
- GBP 30,000 - GBP 37,499 (5)
- GBP 37,500 - GBP 44,999 (6)
- GBP 45,000 - GBP 52,499 (7)
- GBP 52,500 - GBP 59,999 (8)
- GBP 60,000 - GBP 67,499 (9)
- GBP 67,500 - GBP 74,999 (10)
- GBP 75,000 - GBP 99,999 (11)
- GBP 100,000 or more (12)

End of Block: Sociodemographics

# **Figure S1. Distribution of the time taken (in seconds) to complete the survey**

**
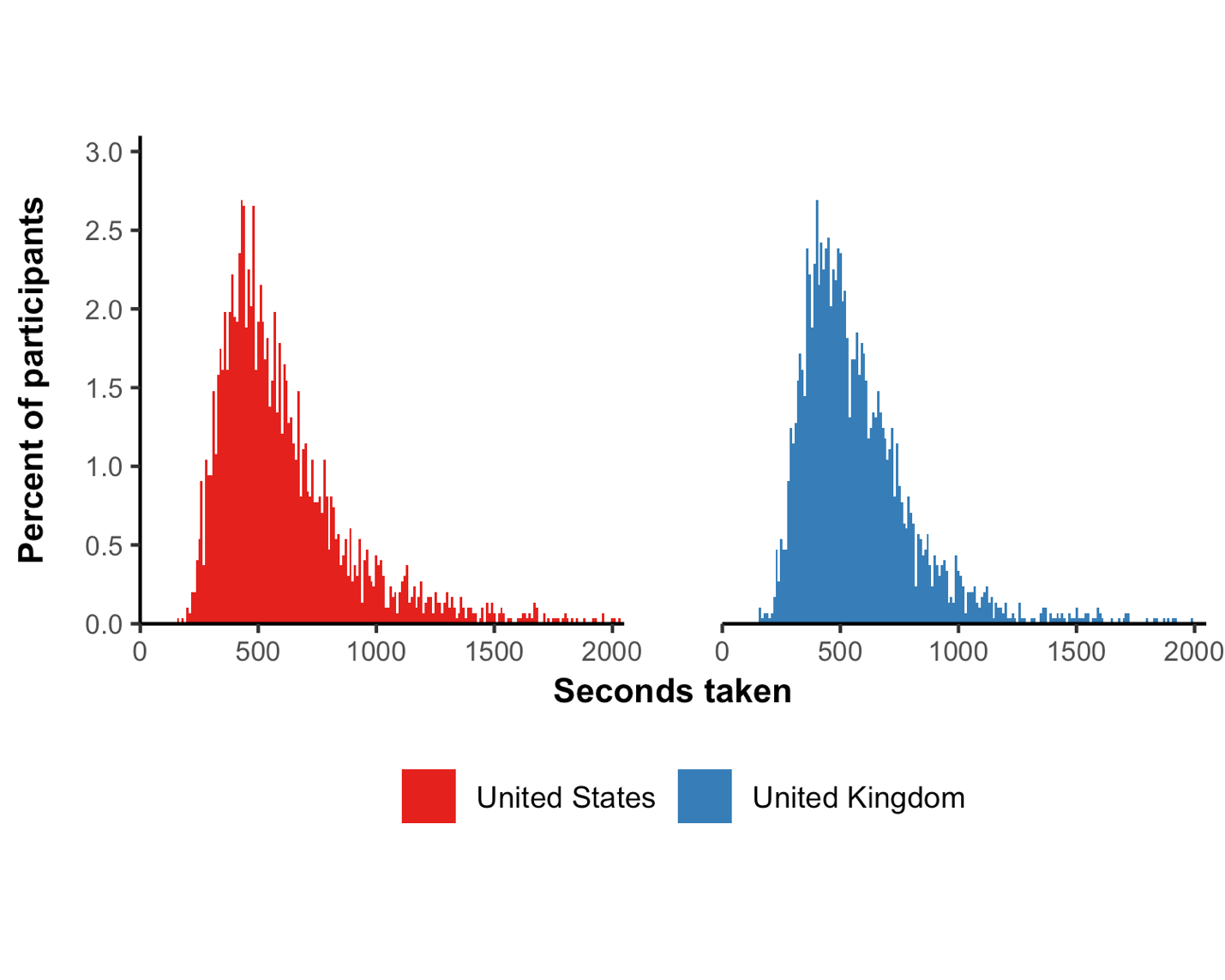
**

# **Figure S2. Distribution of responses to the question “Do you think it is likely that the new coronavirus is a bioweapon developed by a government or terrorist organization?”**

**
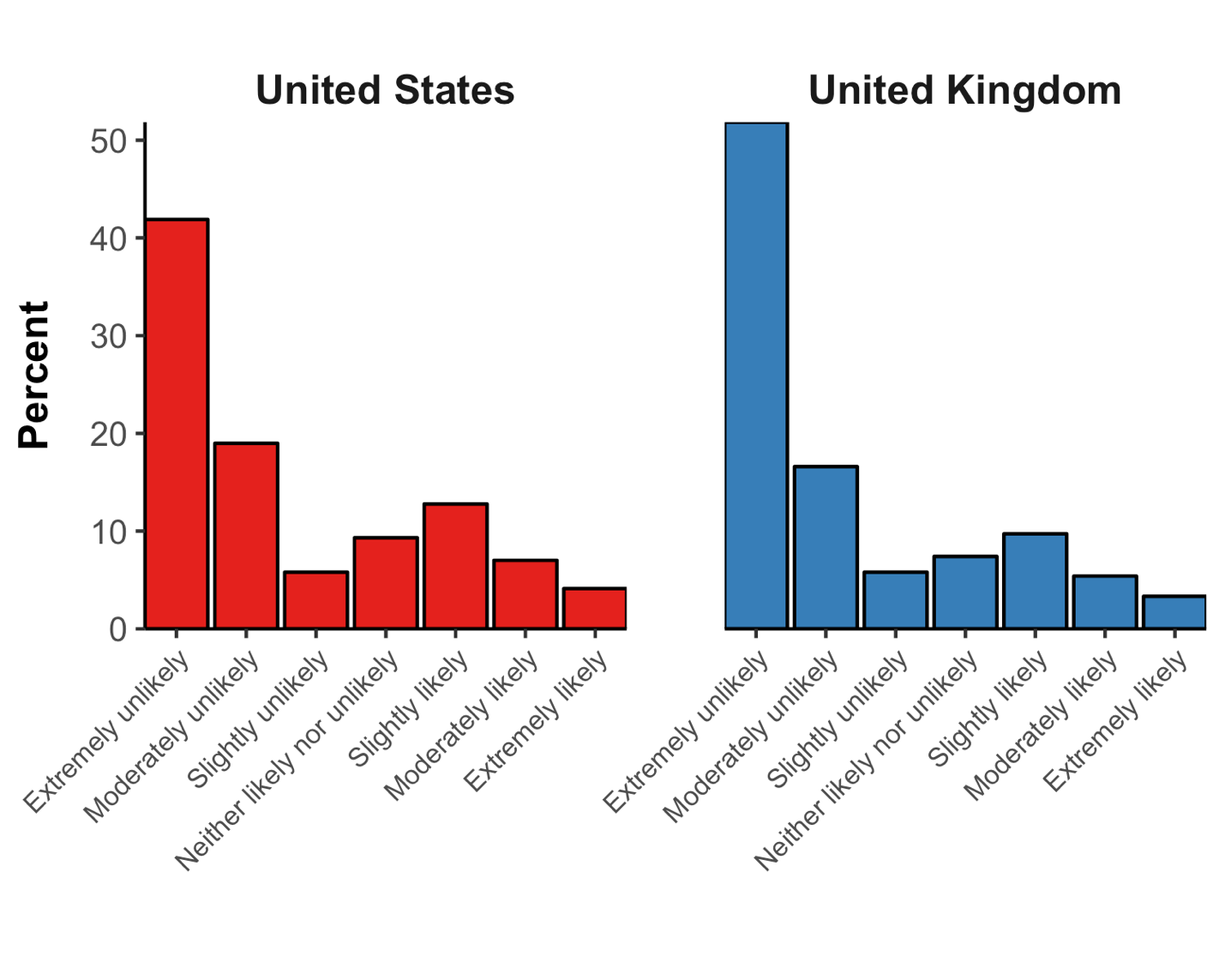
**

# **Figure S3. Distribution of responses to the question “What is the main way in which people are currently getting infected with the new coronavirus? “**

**
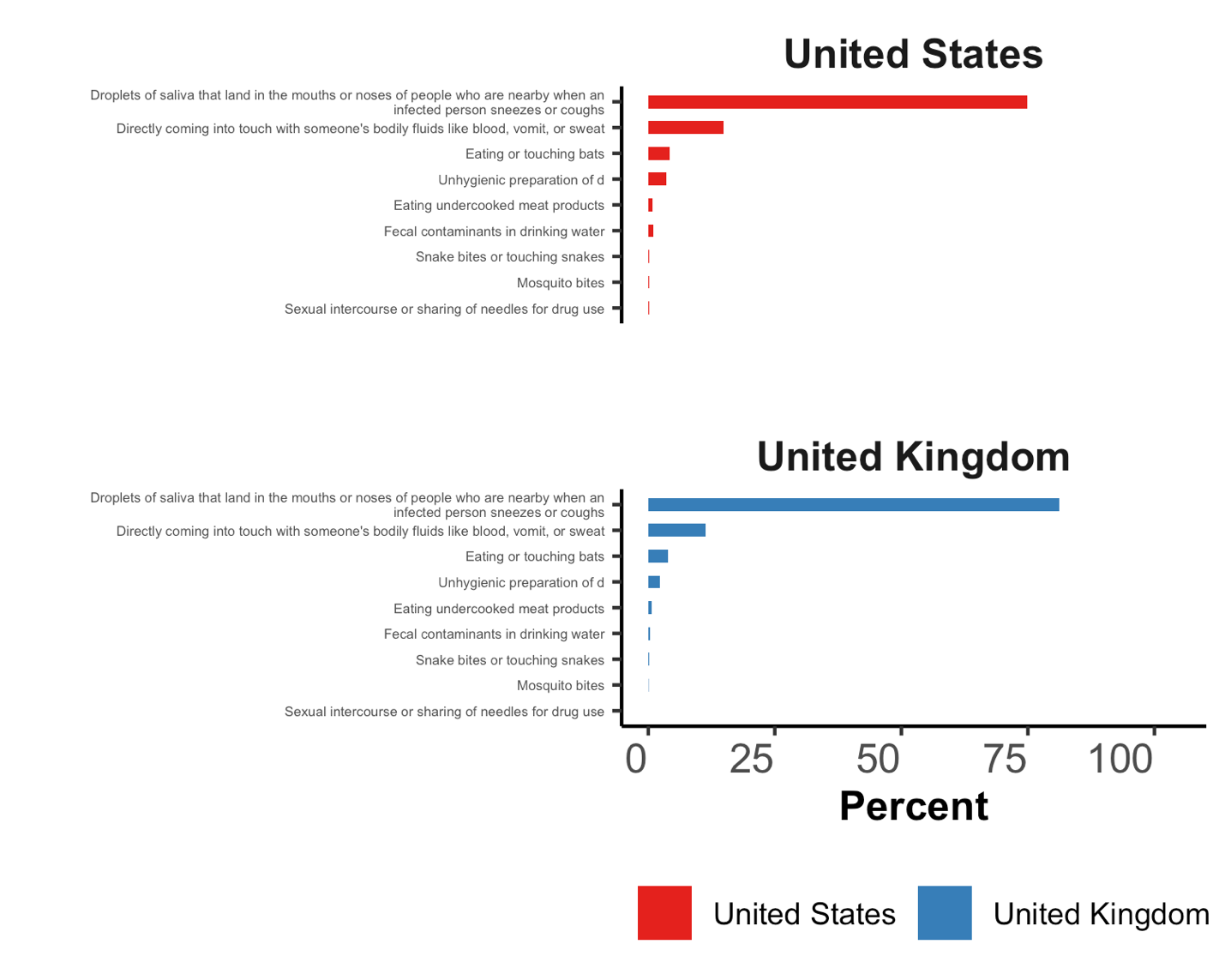
**
